# Vulnerability Factors among Women Victimized by Intimate Partner Violence and the Presence of Children

**DOI:** 10.1007/s10896-021-00328-8

**Published:** 2021-10-18

**Authors:** Joakim Petersson, Sara Thunberg

**Affiliations:** School of Law, Psychology and Social Work, Örebro University, Fakultetsgatan 1, 702 82 Örebro, Sweden

**Keywords:** Intimate partner violence, Victims, Children, Vulnerability factors, Police

## Abstract

This study aimed to a) examine the presence of children in relation to victim vulnerability factors and assessed risk for intimate partner violence (IPV) re-victimization, and b) examine the police response, in terms of risk management, in IPV cases with and without children, respectively. Data from a sample of 1407 women who had reported IPV victimization to the Swedish police was analyzed. The material consisted of risk assessments conducted by the police using the Swedish version of the Brief Spousal Assault Form for the Evaluation of Risk (B-SAFER) checklist, as well as the recommended risk management strategies. A series of chi-square tests of independence revealed that women with and without children, respectively, displayed different vulnerability factors to different extents. Women with children expressed more extreme fear of the perpetrator and were more likely to have an unsafe living situation, whereas women without children displayed more inconsistent attitudes or behaviors and health problems. However, binary logistic regression analyses showed that the victim vulnerability factors that were most strongly associated with an elevated risk rating for IPV re-victimization were generally the same for both groups of victims. Finally, the presence of children was related to a higher risk rating for imminent IPV re-victimization and to recommendations of more than standard levels of risk management strategies. The results indicate that the Swedish police consider the presence of children in relation to a victim’s risk for re-victimization as well as in terms of recommended risk management strategies.

Intimate partner violence (IPV) is a global problem affecting millions of people every day and has been referred to as a public health problem of pandemic proportions (World Health Organization [WHO], 2013). In accordance with the Istanbul convention, IPV can be defined as any form of physical, sexual, psychological, or economic harm or suffering perpetrated by a current or former partner (Council of Europe, 2011). Globally, it is estimated that every third woman has suffered physical or sexual victimization by a current or former partner (WHO, 2013) and that nearly 40% of all homicides with female victims are IPV-related (Stöckl et al., 2013). Corresponding estimates in Sweden, where this study was conducted, show that 25% of women aged 16–79 have reported experiencing IPV victimization (National Council for Crime Prevention [NCCP], 2014). Moreover, 13 women were killed by a current or former intimate partner during 2020 in Sweden (NCCP, 2021).

In their duty to prevent crimes, the police are one of the primary responders to IPV and the first contact for both victims and perpetrators with the criminal justice system (Erez, 2002; Storey et al., 2014). To this end, a widely used prevention strategy adopted by police is the use of violence risk assessments (e.g., Campbell et al., 2018). This is a method for criminal justice professionals to manage violent situations, such as police officers responding to IPV-related calls (Kropp, 2008). These assessments allow for estimating the risk that the victim will be re-victimized by examining the perpetrator’s risk factors for violence. In fact, there are several different IPV risk assessment instruments available to the police, and some of these instruments have been developed specifically for the police to use (e.g., Nicholls et al., 2013).

The Brief Spousal Assault Form for the Evaluation of Risk (B-SAFER: Kropp et al., 2010) is a specific IPV risk assessment tool used by the Swedish police, as well as by police forces internationally. It has been developed for the police and according to the structured professional judgment (SPJ) approach to violence risk assessment. In short, the SPJ approach advocates violence prevention by assessing the presence or absence of risk factors for re-victimization in each case, estimating a summary risk rating for re-victimization (e.g., low, moderate, or high risk), and mitigating this risk by implementing protective actions (e.g., restraining orders or emergency telephones for victims). The latter part, referred to as risk management, is what sets the SPJ approach apart from other methods of violence risk assessment (e.g., the actuarial approach: Kropp, 2008). The B-SAFER consists of ten perpetrator risk factors, divided into the nature of IPV (i.e., violent acts, violent threats or thoughts, escalation of violence, violations of court orders, and violent attitudes), as well as the perpetrator’s psychosocial adjustment (i.e., general criminality, relationship problems, employment problems, substance use problems, and mental health problems).

In addition, drawing on research demonstrating the importance of considering victim characteristics in relation to the risk for re-victimization (e.g., Bennett Cattaneo & Goodman, 2005; Capaldi et al., 2012; Kuijpers et al., 2011), the B-SAFER contains victim vulnerability factors. Acknowledging the central role of victim vulnerability factors in understanding IPV victimization is, and has long been, controversial. Identifying factors and characteristics of victims that render them more likely to be repeatedly victimized may be viewed as blaming the victims for their situation. This fear of victim-blaming is understandable considering that this has been a common societal response to IPV (e.g., Bennett Cattaneo & Goodman, 2005; Erez, 2002). However, as previously noted by others, “to fully understand the dynamics of abusive relationships, it will be necessary to look at both partners” (Henderson et al., 1997, p. 187). Importantly, the intended purpose of identifying vulnerability factors using the B-SAFER is to help the police to determine the likelihood that the victim will engage in self-protective actions and, thus, adhere to the recommended risk management strategy (Kropp et al., 2010). For instance, it would be unlikely that a restraining order will be an effective protective action in cases where the victim justifies the perpetrator’s violent behavior and wants to maintain the relationship.

Drawing on the abovementioned, the B-SAFER requires the user to assess the presence or absence of five victim vulnerability factors (Kropp et al., 2010). In line with the ten perpetrator risk factors in the B-SAFER, these vulnerability factors were identified through a systematic review of the IPV re-victimization literature. The first factor is *inconsistent behavior or attitude towards the perpetrator*. This includes behaviors and attitudes that can increase the risk of being re-victimized, such as minimizing or justifying the perpetrator’s use of violence or contacting the perpetrator despite a restraining order being in effect. Reasons for such behavior or attitudes could be explained by a victim’s emotional attachment to the perpetrator, including fear, love, or sympathy for the perpetrator (e.g., Henderson et al., 1997; Kropp et al., 2010). The second factor involves *extreme fear of the perpetrator*, which makes the victim panic and irrational (e.g., such as returning to the abusive relationship out of fear for violence escalation: Kropp et al., 2010). Previous research has demonstrated that a victim’s fear of the perpetrator is related to a higher police-assessed risk for re-victimization (Belfrage & Strand, 2008; Robinson et al., 2018; Trujillo & Ross, 2008). For instance, Trujillo and Ross (2008) found that extreme fear of the perpetrator was the strongest predictor for such risk. More specifically, in IPV cases where victims expressed being fearful of the perpetrator, the police assessed the risk for re-victimization as higher compared to in cases where victims were not fearful. Moreover, a victim’s level of fear was also found to be a significant predictor of police decisions on risk management strategies, where victims who expressed higher levels of fear of the perpetrator were subjected to more protective actions (Trujillo & Ross, 2008).

The third factor in the B-SAFER, *inadequate support or resources*, relates to the victim’s knowledge, motivation, or ability to seek help. This can be complicated by, for example, language barriers, being unaware of one’s legal rights, or the social isolation of the victim (Kropp et al., 2010). The fourth factor relates to the victim’s *unsafe living situation*, which can include remaining living with the perpetrator or lacking sufficient protection in the workplace (Kropp et al., 2010). Finally, the fifth vulnerability factor refers to the victim’s *health problems* that may have been developed through the course of victimization or problems that may have preceded the victimization. This can include problems related to substance use or mental health, but also to unemployment as this can create psychosocial problems such as depression and stress (Kropp et al., 2010). These health problems can make the victim dependent on the perpetrator, for example in relation to the access to alcohol, drugs, or income, and thereby make victims reluctant to leave an abusive relationship. Thus, the B-SAFER victim vulnerability factors pertain to the victim’s behavioral, psychosocial, and situational vulnerabilities, which directly or indirectly may increase the risk of being re-victimized by IPV.

Previous research has found that the B-SAFER victim vulnerability factors influence police decisions on risk assessment and management. As such, Belfrage and Strand (2008) demonstrated that the presence of vulnerability factors was positively correlated to a higher assessed risk for IPV re-victimization. More importantly, they concluded that the inclusion of vulnerability factors in risk assessment instruments was welcomed by the police, who perceived these to allow for a more complete and holistic assessment of risk for IPV re-victimization. Storey and Strand (2017) found that police recommendation of risk management strategies were consistently related to the presence of vulnerability factors and summary risk ratings for female victims (i.e., more protective actions were recommended in cases with more vulnerability factors present as well as in cases with a higher summary risk rating). Finally, Strand and Storey (2019) reported that victims living in rural and remote areas had more vulnerability factors present than victims living in urban areas and that the severity of violence was higher in rural and remote areas.

Still, less is known about how victim vulnerability factors may differ in relation to other important factors that can influence the risk for re-victimization and police officers’ decisions relating to risk assessment and management. To this end, several authors have emphasized the need to study the presence of children. Considering the global estimates indicating that between 133 and 275 million children witness parental IPV every year (United Nations, 2006), it is surprising that the presence of children is rarely studied in terms of risk for IPV. Instead, studies have mainly focused on childhood abuse as a risk factor for IPV perpetration and victimization (Bennett Cattaneo & Goodman, 2005; Capaldi et al., 2012; Yakubovich et al., 2018). Among the few studies conducted on the presence of children as a predictor for IPV re-victimization, the results have been inconclusive (Bennett Cattaneo & Goodman, 2005). Furthermore, drawing on the limited body of knowledge Capaldi et al. (2012) concluded their systematic review of risk factors for IPV with the recommendation to study the role that the presence of children in the relationship plays. Thus, little is known about how the presence of children may influence the risk for IPV re-victimization, but also whether victims with children display different victim vulnerability factors than victims without children.

There are several ways in which the presence of children may influence a victim’s vulnerabilities, as well as the risk for re-victimization. To this end, the limited number of studies identified has focused on female victims of IPV. Witnessing parental IPV or being abused as a child within the family can result in several internalizing and externalizing consequences among children such as depression, posttraumatic stress, behavioral and relationship problems (Cater et al., 2015; Holt et al., 2008), rendering these children more difficult to parent. Additionally, being victimized by IPV can negatively affect a woman’s parenting skills and ability to nurture her children as IPV victimization often is associated with mental health problems, lack of self-esteem, and feeling disempowered (Anderson & Van Ee, 2018; Letourneau et al., 2011). Thus, due to the negative effects of being victimized by IPV, women might find it difficult to handle their children’s externalizing and internalizing problems.

The presence of children is also a factor that can both enable and hinder the woman from leaving the violent relationship. On the one hand, women describe that they want to keep their families together, that they are afraid of the unknown, afraid of the justice system, and especially afraid that the social services will take their children from them (Rhodes et al., 2010; Zink et al., 2003). Moreover, the presence of children has been regarded by the victims as a practical obstacle for leaving the perpetrator (e.g., Estrellado & Loh, 2014). As such, women who have invested more time and resources into the relationship (e.g., having children with the perpetrator) may be more reluctant to leave. On the other hand, the presence of children can empower women to leave, as they realize that the violence affects the children negatively, that the children need protection, or that the children might lose their mother due to the violence (e.g., Rhodes et al., 2010; Zink et al., 2003). Thus, the presence of children has been found to be an important factor that affects women’s decision to leave the abusive relationship, and if they seek help from the criminal justice system.

Relatedly, little is known about how the presence of children may influence police decisions on risk assessment and management of IPV (Saxton et al., 2020). Children are often referred to as ‘the forgotten victims’ as their presence is often overlooked by the police (Reif & Jaffe, 2019). For example, one study reported that police officers were inclined to view children as passive observers rather than indirect victims of IPV (Richardson-Foster et al., 2012). Moreover, Saxton et al. (2020) described the police response, with regards to risk assessment and management of IPV cases with children present, as inconsistent and in need of future scrutiny.

## The Present Study

Despite their potential influence, the presence of children has rarely been examined in relation to risk for re-victimization among victims of IPV, or in relation to the victim’s vulnerability (Bennett Cattaneo & Goodman, 2005; Capaldi et al., 2012; Yakubovich et al., 2018). Moreover, few IPV risk assessment instruments include the presence of children as a risk factor for re-victimization (Nicholls et al., 2013). Drawing on previous recommendations of advancing the knowledge of what role children play in partner violent relationships (e.g., Capaldi et al., 2012; Saxton et al., 2020), this study seeks to address several knowledge gaps related to how the presence of children may impact victim vulnerability factors and the risk for re-victimization. First, although previous studies have examined differences in the presence of the B-SAFER vulnerability factors in relation to victim gender and rurality (Storey & Strand, 2017; Strand & Storey, 2019), no study has examined such differences in relation to the presence of children. Such knowledge could help identify any specific needs that may differ between victims with children and victims without children. For instance, victims with children may require different services and support, given that their children are also being exposed to violence. Second, the literature examining the presence of children as a risk factor for IPV re-victimization is both scarce and inconclusive (Bennett Cattaneo & Goodman, 2005; Capaldi et al., 2012; Yakubovich et al., 2018). To this end, this study will add to the existing literature by examining the police-assessed risk for IPV re-victimization, as well as examine the relative importance of specific vulnerability factors for such risk, among victims with children and victims without children. Finally, heeding the call for more research in relation to the police response to IPV cases with children present (Saxton et al., 2020), this paper will also examine the police response to IPV cases with and without children, respectively, in terms of the recommended risk management.

Thus, the aim of this study was twofold. First, to examine the presence of children in relation to victim vulnerability factors and assessed risk for IPV re-victimization. Second, to examine the police response, in terms of risk management, in IPV cases with and without children, respectively. More specifically, this study sought to address the following research questions:

1. How do victims with and without children, respectively, differ in terms of vulnerability factors?
2. How do victims with and without children, respectively, differ in terms of assessed risk for being re-victimized, and which victim vulnerability factors are most strongly associated with such a risk?
3. How do cases with children differ from cases without children in terms of the number and type of risk management strategies recommended by the police?

## Method

### Sample

The sample in this study was drawn from an eight-year (2009–2016) prospective research project conducted in collaboration with the Swedish police. More specifically, this project aimed to implement and evaluate the use of structured violence risk assessments for various forms of family violence in four Swedish police districts (Strand et al., 2016). The police officers conducting the risk assessments and recommending the risk management were all working within family violence units within the police. Thus, these police officers were specially trained in IPV risk assessment and management (i.e., both in theory and practice).

Initially, the sample consisted of 1573 female victims of IPV, whose male partner was reported to the police in any of the four police districts between 2009 and 2014 for an IPV-related crime. Cases with missing information about children (*n* = 86, 5.5%) or with victim vulnerability factors missing completely (i.e., not assessed at all: *n* = 80, 5.1%) were excluded. Thus, the final sample in this study consisted of 1407 female victims of IPV. Cases where vulnerability factors were left completely unassessed did not differ significantly from the remaining cases where at least one vulnerability factor was assessed, in terms of the presence of children or assessed risk for re-victimization.

Demographical information about the 1407 victims was only available for a subset of the total sample (*N* = 983). Based on this subset, the mean age of the sample was 35.9 years (*SD* = 12.5; *Range* = 14–83), and 20.4% of the victims had an immigrant background. Most police reports consisted of physical assault (48.9%), followed by severe violation of a woman’s integrity (25.2%), threats (13.3%), other crimes (e.g., harassment or stalking: 5.1%), sexual assault (1.5%), and attempted murder/manslaughter (0.5%). Eighty-one (5.4%) cases were missing information about the type of crime reported. The crime of severe violation of a woman’s integrity is internationally unique for Swedish legislation, where male perpetrators who are found guilty of repeatedly committing crimes (i.e., on more than one occasion and towards the same woman) aimed at violating their intimate female partner’s integrity can be charged with ‘severe violation of a woman’s integrity’. The purpose of this crime is that it allows the court to sentence the perpetrator to a harsher sentence, taking the woman’s overall victimization into account.

### Material

The material consisted of structured violence risk assessments carried out by the police, using the Swedish version of the B-SAFER (Kropp et al., 2010), as well as additional case-related information collected by the police (e.g., the presence of children). The risk and victim vulnerability factors in the B-SAFER are scored on a three-point scale as either absent (No), partially present (To some extent), or present (Yes). This scoring procedure should be based on hearings with the perpetrator, the victim, and any potential witnesses, as well as on data available from police registers. Based on the presence or absence of the risk and victim vulnerability factors, the assessor makes two summary ratings of overall risk for future violence: one pertaining to the risk for imminent violence, and one pertaining to the risk for severe or lethal violence. These summary risk ratings are assessed as low, moderate, or high risk (Kropp et al., 2010).

Based on the summary risk ratings for re-victimization generated from assessing the risk and vulnerability factors, the police should also suggest adequate protective actions to reduce this risk. Therefore, the risk management strategies that the police can recommend in each B-SAFER assessment were also analyzed. The risk management strategies were categorized as standard level of risk management and more than standard level of risk management. Standard risk management, which is mandatory for the police to carry out when conducting safety planning in IPV cases, includes searching crime registers for information about the perpetrator (e.g., previous criminal history), and referrals to crime victim support services. More than standard level of risk management included the following protective actions: initiating contact with a legal counsel for the victim, initiating an application for a restraining order, protecting the victim’s identity (by concealing information about a person, such as name, social security number, and address, from official registers), holding a security talk with the victim about their situation (e.g., the risk for re-victimization), providing the victim with alarm package or emergency telephone, protected living (e.g., at a women’s shelter), or referring the case to a specialized police unit, dealing with high-risk cases, for personal security. However, as the police did not systematically document which of the recommended protective actions were carried out, we only had information about what was recommended in each case.

In addition to the B-SAFER assessment, the police also filled in background information about each case, including the presence of children. To this end, the available information about the presence of children was limited to whether children younger than 18 years were living at home with the victim. No other systematically collected data related to children’s exposure to IPV was available (e.g., witnessing parental IPV or being abused themselves). The reported IPV crime was analyzed to control for violence severity. All crimes involving any physical violence (e.g., assault) were categorized as more severe, whereas non-physical crimes were categorized as less severe. This categorization of severity emanated from the risk of physical injuries to the victim. However, it should be noted that non-physical IPV can be perceived by victims as being equally severe in its consequences as physical IPV (e.g., O’Leary, 1999).

### Procedure

Upon receiving reports of IPV cases, the police first filled out background information about the case (e.g., demographics of the victim and perpetrator, as well as the presence of children), including a brief description of the IPV incident. Second, the ten perpetrator risk factors in the B-SAFER were assessed. Third, the five victim vulnerability factors in the B-SAFER were assessed. Fourth, drawing on the presence and absence of the risk and victim vulnerability factors in each case, the police provided two summary risk ratings for the risk of recidivism (i.e., both for imminent risk and for severe forms of IPV). Fifth, based on the outcome of the B-SAFER assessment, the police suggested various protective actions (i.e., risk management strategies) to be initiated in each case. Data were collected at the police stations.

### Statistical Analyses

In comparing victims with children and victims without children on categorical data, chi-square tests (χ^2^) of independence, with Cramer’s V as a measure of effect size, were used. To further examine the differences in relation to categorical data between the two groups of victims, odds ratios (OR) with 95% confidence intervals (CI) were also used. OR greater than 1 indicate an increase in odds of the outcome, whereas OR less than 1 indicate a decrease in odds of the outcome measured. Comparisons between the two groups on ratio level data were calculated using independent samples *t*-test. To examine the relative importance of each victim vulnerability factor to the summary risk ratings for re-victimization (i.e., the risk for imminent re-victimization and risk for severe or lethal IPV), a series of binary logistic regression analyses were carried out. In these analyses, the victim vulnerability factors were entered as predictors, using the summary risk ratings as dependent variables.

To enable OR and logistic regressions, and for ease of interpretation, the victim vulnerability factors, originally assessed on an ordinal level (i.e., present, partially present, and absent), were collapsed into dichotomous variables measuring the presence (i.e., combining ratings of present and partially present) and absence of such factors. Furthermore, the summary risk ratings of imminent IPV and severe or lethal IPV were also dichotomized as elevated risk (i.e., combining ratings of moderate and severe risk) and low risk. The dichotomization of the B-SAFER risk factors and summary risk ratings has previously been done (e.g., Petersson et al., 2019). All statistical analyses were carried out in IBM SPSS version 26. This study has received ethical approval from the Swedish Ethical Review Authority.

## Results

Preliminary analyses showed that children younger than 18 years were present in more than half (56.8%) of all IPV cases reported to the police. Moreover, women with children were younger (*M* = 34.0, *SD* = 8.2) than victims without children (*M* = 37.5, *SD* = 15.7), *t*(523) = 3.71, *p* = 0.000. In terms of violence severity, women without children were twice as likely as women with children to have been victimized by more severe forms of IPV (i.e., physical violence: 86.5% vs 76.0%), χ^2^ (1, 1331) = 22.78, *p* = 0.000, *phi* = -0.13 (OR = 2.0; 95% CI = [1.5, 2.7]).

For the first set of analyses, women with and without children, respectively, were compared in terms of the number and type of victim vulnerability factors present. An independent samples *t*-test revealed no significant difference between women with children (*M* = 2.2, *SD* = 1.4) and women without children (*M* = 2.2, *SD* = 1.6) in terms of the mean number of victim vulnerability factors assessed by the police as being present, *t*(1222) = 0.16, *p* = 0.870. Differences between the two groups in terms of the type of vulnerability factors present are shown in Table 1. As such, women with and without children, respectively, differed significantly on all vulnerability factors but inadequate support or resources. Women with children were more likely to have expressed extreme fear of the perpetrator and having an unsafe living situation. However, this group was less likely to display inconsistent attitudes or behavior towards the perpetrators as well as less likely to display health problems. Noteworthy, however, the presence of the B-SAFER vulnerability factors was high in the overall sample.

**Table 1:** Presence of victim vulnerability factors among IPV victims with or without children (N = 1407)

B-SAFER item	Present							
	Missing (%)	Children (*n* = 801)		No children (*n* = 606)				
Victim vulnerability factors		*N*	*%*	*N*	%	*χ*^*2*^	Cramer’s V	OR (95% CI)
Inconsistent attitudes or behavior	10.5	401	56.2	350	64.2	8.34**	.08	1.4 [1.1, 1.8] ^†^
Extreme fear of perpetrator	10.4	382	52.2	234	44.3	7.60**	.08	1.4 [1.1, 1.7]
Inadequate support or resources	14.3	253	36.9	190	36.5	0.02	.00	
Unsafe living situation	16.0	460	68.6	279	54.6	24.11***	.14	1.8 [1.4, 2.3]
Health problems	26.7	262	46.5	285	61.0	21.79***	.14	1.8 [1.4, 2.3] ^†^

*Notes*. IPV = Intimate partner violence; B-SAFER = Brief Spousal Assault Form for the Evaluation of Risk (Kropp et al., 2010). OR = odds ratios. CI = confidence intervals

^*^p < .05. **p < .01. ***p < .001

^†^ Odds ratios inverted (1/OR) for ease of interpretation

Risk for re-victimization among women with or without children was measured using the two summary risk ratings in the B-SAFER. In terms of risk for imminent re-victimization, a chi-square test of independence demonstrated a significant difference between the proportion of women with (61.3%) and without children (53.9%), respectively, who were assessed with an elevated risk for being re-victimized, χ^2^ (1, 1180) = 6.53, *p* = 0.011, *phi* = 0.07 (OR = 1.3; 95% CI = [1.1, 1.7]). Thus, women with children were more likely to be assessed with an elevated risk for imminent re-victimization compared to women without children. In terms of risk for severe or lethal violence, no significant difference was found for the proportion of women with (39.8%) or without children (37.5%) who were assessed with an elevated risk for such violence, χ^2^ (1, 1381) = 0.78, *p* = 0.376, *phi* = 0.02 (OR = 1.1; 95% CI = [0.9, 1.4]).

Subsequently, several binary logistic regression analyses were carried out to examine which victim vulnerability factors were most strongly associated with an elevated risk rating for imminent IPV, as well as severe or lethal IPV, within the two groups of victims. Table 2 displays the results related to vulnerability factors that predicted an elevated risk rating for imminent IPV re-victimization among women with and without children, respectively. Among women with children, inconsistent attitudes or behavior (OR = 1.7), extreme fear of the perpetrator (OR = 2.3), and an unsafe living situation (OR = 3.2) significantly predicted an elevated risk for imminent IPV re-victimization. Having an unsafe living situation was the strongest predictor, as women with this vulnerability factor were more than three times as likely to be assessed with an elevated risk for being re-victimized. Similarly, among women without children, extreme fear of the perpetrator (OR = 2.5), inadequate support or resources (OR = 1.9), and unsafe living situation (OR = 3.2) significantly predicted an elevated risk for imminent IPV re-victimization.

**Table 2:** Logistic regression models of the B-SAFER victim vulnerability factors’ predictability of elevated summary risk ratings for imminent re-victimization among female IPV victims with and without children, respectively

Children present (*n* = 376)	β	Wald	*p*	*OR*	95% CI
Model^a^					
Inconsistent attitudes or behavior	0.5	4.6	.033	1.7	[1.1, 2.7]
Extreme fear of perpetrator	0.8	11.3	.001	2.3	[1.4, 3.7]
Inadequate support or resources	0.5	3.5	.061	1.7	[1.0, 3.0]
Unsafe living situation	1.1	18.7	.000	3.2	[1.8, 5.4]
Health problems	0.3	1.1	.292	1.3	[0.7, 2.3]
Constant	-1.4	31.7	.000		
No children (*n* = 308)	β	Wald	*p*	*OR*	95% CI
Model^b^					
Inconsistent attitudes or behavior	0.1	0.2	.685	1.1	[0.6, 2.0]
Extreme fear of perpetrator	0.9	11.2	.001	2.5	[1.5, 4.3]
Inadequate support or resources	0.6	3.9	.049	1.9	[1.0, 3.6]
Unsafe living situation	1.2	17.6	.000	3.2	[1.8, 5.4]
Health problems	0.2	0.6	.447	1.3	[0.7, 2.3]
Constant	-1.2	27.0	.000		

*Notes.* B-SAFER = Brief Spousal Assault Form for the Evaluation of Risk (Kroppet al., 2010). IPV = Intimate partner violence. OR = Odds ratios. CI = confidence interval. Summary risk ratings dichotomized as ‘low risk’ (i.e., low risk) and ‘elevated risk’ (i.e., moderate or high risk). Due to missing values, two sub-samples (*n* = 376 and *n* = 308) with complete coding for summary risk ratings of imminent IPV in the B-SAFER were included for analyses

^a^ Omnibus tests of model coefficients = χ^2^ (5) = 74.91, *p* = .000. Cox & Snell R square = .181. Nagelkerke R square = .242

^b^ Omnibus tests of model coefficients = χ^2^ (5) = 71.33, *p* = .000. Cox & Snell R square = .207. Nagelkerke R square = .276

Table 3 displays the results of predictors for an elevated risk for severe or lethal IPV among women with and without children, respectively. For women with children, extreme fear of the perpetrator (OR = 3.9) and inadequate support or access to resources (OR = 3.2) significantly predicted an elevated risk for severe or lethal IPV, increasing the risk by nearly four and three times, respectively. Among women without children, extreme fear of the perpetrator (OR = 3.2) and an unsafe living situation (OR = 3.2) were significant predictors for an elevated risk for severe or lethal IPV, increasing this risk by more than three times if present.

**Table 3:** Logistic regression models of the B-SAFER victim vulnerability factors’ predictability of elevated summary risk ratings for severe or lethal re-victimization among female IPV victims with and without children, respectively

Children present (*n* = 457)	β	Wald	*p*	*OR*	95% CI
Model^a^					
Inconsistent attitudes or behavior	0.1	0.1	.806	1.3	[0.6, 2.5]
Extreme fear of perpetrator	1.0	22.4	.000	3.9	[2.0, 7.7]
Inadequate support or resources	0.5	4.4	.037	3.2	[1.4, 7.2]
Unsafe living situation	0.4	2.7	.103	1.6	[0.7, 3.6]
Health problems	0.2	0.8	.377	1.0	[0.5, 2.2]
Constant	-1.6	51.7	.000		
No children (*n* = 368)	β	Wald	*p*	*OR*	95% CI
Model^b^					
Inconsistent attitudes or behavior	0.1	0.1	.810	1.1	[0.6, 1.9]
Extreme fear of perpetrator	1.2	22.2	.000	3.2	[2.0, 5.3]
Inadequate support or resources	-0.1	0.1	.753	0.9	[0.5, 1.6]
Unsafe living situation	1.2	18.4	.000	3.2	[1.9, 5.4]
Health problems	0.5	3.1	.077	1.7	[0.9, 2.9]
Constant	-2.0	60.6	.000		

*Notes.* B-SAFER = Brief Spousal Assault Form for the Evaluation of Risk (Kroppet al., 2010). IPV = Intimate partner violence. OR = Odds ratios. CI = confidence interval. Summary risk ratings dichotomized as ‘low risk’ (i.e., low risk) and ‘elevated risk’ (i.e., moderate or high risk). Due to missing values, two sub-samples (*n* = 457 and *n* = 368) with complete coding for summary risk ratings of severe/lethal IPV in the B-SAFER were included for analyses

^a^ Omnibus tests of model coefficients = χ^2^ (5) = 54.20, *p* = .000. Cox & Snell R square = .112. Nagelkerke R square = .152

^b^ Omnibus tests of model coefficients = χ^2^ (5) = 75.94, *p* = .000. Cox & Snell R square = .186. Nagelkerke R square = .253

Finally, the recommended risk management strategies for women with children and women without children were analyzed. However, such information was only available for a subset of the sample (*N* = 980). A chi-square test of independence revealed a significant difference in terms of the relationship between levels of recommended risk management strategies and the presence of children. As such, the police were more likely to recommend more than standard levels of risk management in cases with children (71.3%) than in cases without children (62.2%), χ^2^ (1, 956) = 8.84, *p* = 0.003, *phi* = 0.01 (OR = 1.5; 95% CI = [1.2, 2.0]). More specifically, as can be seen in Table 4, the police were more likely to recommend that women with children apply for a restraining order (OR = 1.6) and protecting a victim’s identity (OR = 2.8). In addition, the police were more likely to provide women with children an emergency telephone (OR = 2.5).

**Table 4:** Risk management strategies recommended by the police in IPV cases with or without children (N = 980)

Present								
	Missing (%)	Children		No children				
Risk management strategy		*N*	%	*N*	%	*χ*^*2*^	Cramer’s V	OR (95% CI)
Legal counsel for victim	5.6	203	42.4	177	39.7	0.69	.03	
Restraining order	6.5	222	46.6	153	34.8	13.32***	.12	1.6 [1.3, 2.2]
Protecting a victim’s identity	9.6	20	4.4	7	1.6	5.70*	.08	2.8 [1.2, 6.6]
Security talk with victim	9.9	123	27.2	99	23.0	2.00	.05	
Emergency telephone	10.4	23	5.1	9	2.1	5.72*	.08	2.5 [1.2, 5.5]
Alarm package	10.2	22	4.9	14	1.6	1.50	.04	
Protected living	10.2	44	9.8	31	7.2	1.81	.04	
Referred to specialized police unit for personal security	10.3	15	3.3	11	2.6	0.44	.02	

*Notes*. IPV = Intimate partner violence; B-SAFER = Brief Spousal Assault Form for the Evaluation of Risk (Kropp et al., 2010). OR = odds ratios. CI = confidence intervals

^*^p < .05. **p < .01. ***p < .001

## Discussion

The aim of this study was, firstly, to examine the presence of children in relation to victim vulnerability factors and assessed risk for re-victimization. Secondly, this study also aimed to examine the police response, in terms of recommended risk management, in cases with and without children, respectively. In line with previous findings of IPV reported to the police, children were present in more than half of all IPV cases (e.g., Fantuzzo & Fusco, 2007). Overall, the results showed that the two groups of women displayed different vulnerability factors to a different extent. However, there were generally few differences between the two groups in terms of which vulnerability factors contributed most to an elevated assessed risk for being re-victimized. Finally, victims with children were assessed by the police with a higher risk for imminent re-victimization and were recommended more than standard levels of risk management strategies.

In general, the results demonstrated a high prevalence of vulnerability factors among the women in our sample. Therefore, women with and without children, respectively, did not differ in terms of the mean number of vulnerability factors present. This could be explained by the fact that the IPV reported to the police is usually more severe (NCCP, 2014; Sartin et al., 2006). More importantly, however, women with and without children, respectively, displayed different types of vulnerabilities. For women with children, extreme fear of the perpetrator and having an unsafe living situation were more common. The higher prevalence of extreme fear of the perpetrator may relate to also fearing for their children’s physical and emotional safety and well-being (Rhodes et al., 2010; Zink et al., 2003). Furthermore, drawing on the increased likelihood of the co-occurrence of child abuse in relationships with IPV (e.g., Fernández-González et al., 2018), it is possible that the fear expressed by women with children in this study could be because her children were also victimized.

Another possible source of the extreme fear of the perpetrator expressed by women with children could be their unsafe living situation. As such, the presence of children could indicate that the victim and the perpetrator are more likely to be living together or staying in contact even after separation, for example, due to joint child custody (e.g., Carlson et al., 1999; Laing, 2017). However, even if the woman after separating from the perpetrator has sole custody, the woman and her children may still have an unsafe living situation, as the perpetrator still might have knowledge about their daily whereabouts and routines, which can increase the risk for re-victimization. To this end, studies show that partner violent men often use children instrumentally, through joint custody or through custody disputes, to continue to abuse and control their former partner (e.g., Laing, 2017). In contrast, women without children may be less likely to live together with the perpetrator and are not forced to keep in contact after separation.

This study also found that women without children were more likely to display inconsistent attitudes or behavior towards the perpetrator and having more health problems. It is reasonable to believe that these vulnerability factors might interact. As such, health problems (e.g., substance abuse or unemployment) can make victims dependent on their perpetrator (e.g., for drugs or money) and, therefore, decide to maintain the abusive relationship (e.g., Estrellado & Loh, 2014). Thus, the IPV may be reported to the police with the aim of ending the violence, but does not necessarily mean that the victim can, or wants to, end the relationship. In turn, this could be perceived by the police as a display of inconsistent attitudes or behavior from the victim (i.e., reporting the perpetrator while maintaining the relationship). Another explanation for the higher frequency of health problems among women without children may be related to this group being older than women with children. As such, given that both physical and mental health problems tend to increase with age, this could perhaps partially explain the group differences. Problematically, ‘health problems’ was not further specified in the data collected for this study. Thus, we do not know what specific health problems women displayed, and any conclusions should be made with caution.

Meanwhile, the rate of health problems was high also among women with children, where nearly half of these women displayed such problems. Again, this is not surprising considering that IPV victimization can result in several physical, psychological, and financial problems (e.g., WHO, 2015), coupled with the higher violence severity of IPV reported to the police (e.g., Sartin et al., 2006). Drawing on previous research, it is possible that such health problems (e.g., mental health problems) may stem from the women’s difficulties of handling their children’s externalizing and internalizing problems, which may have been caused by the children’s exposure to IPV (Anderson & Van Ee, 2018; Cater et al., 2015). Once again, however, our lack of more detailed data regarding the vulnerability factors limits our ability to draw any certain conclusions. Also, women with children displayed a high prevalence of inconsistent attitudes or behavior (56.2%). Having children with the perpetrator makes it more difficult to leave the relationship as victims may consider this as a practical obstacle for leaving the abusive relationship (Estrellado & Loh, 2014; Rhodes et al., 2010; Zink et al., 2003). Thus, a decision to stay could be interpreted by the police as displaying inconsistent attitudes or behaviors.

Findings in relation to the risk for being re-victimized by IPV were mixed, where women with children were deemed to be more likely to be re-victimized within the nearest future (i.e., imminent), but not to be at a higher risk for being victimized by severe or lethal forms of IPV. As such, these women may still live together with the perpetrator or are forced to keep in contact due to joint custody or custody disputes (Carlson et al., 1999). Due to this contact, the risk for imminent IPV re-victimization may be assessed as higher by the police. The police may also perceive children as potential targets of the violence, and therefore assess this risk as higher. However, to our knowledge, no study has previously examined the police-assessed risk for IPV re-victimization in relation to the presence of children and more research is needed before any firm conclusions can be made. Finally, although women without children were more likely to have been victimized by severe forms of IPV, there was no difference in terms of assessed risk for severe or lethal IPV between the two groups of victims. These findings indicate that neither the presence of children nor prior violence severity are decisive factors for the police when assessing the severity of any possible re-victimization. To gain a deeper understanding of what factors the police regard as central in assessing such a risk, this research area could benefit from more qualitative studies (see for example Robinson et al., 2018).

In terms of specific victim vulnerability factors that increased the risk of being re-victimized, there were few differences between the two groups. Overall, extreme fear of the perpetrator and an unsafe living situation were more pertinent for an elevated assessed risk for both imminent and severe or lethal IPV re-victimization among both groups (cf. Belfrage & Strand, 2008; Robinson et al., 2018). These results indicate that the police deem these vulnerability factors as most important in relation to risk for IPV re-victimization among victims in general and, thus, do not differentiate between victims with and without children, respectively. However, the results concerning the victim’s fear of the perpetrator is in line with previous studies (Belfrage & Strand, 2008; Robinson et al., 2018; Trujillo & Ross, 2008). As such, Trujillo and Ross (2008) found that extreme fear of the perpetrator was the strongest predictor for the police when assessing a case as having a high risk for re-victimization. The influence of a victim’s fear of the perpetrator on police assessments of risk for re-victimization could be explained with that such fear signals a higher legitimacy of the reported accusation, higher severity of the IPV, an indicator of a history of abuse, or the perpetrator’s capacity for using severe forms of violence (Robinson et al., 2018; Trujillo & Ross, 2008). Concerning the importance of an unsafe living situation, a possible explanation is that such a situation indicates that the perpetrator has access to the victim and that the victim lacks the protection necessary to prevent re-victimization (Kropp et al., 2010).

Some vulnerability factors uniquely contributed to an elevated risk rating for IPV re-victimization among women with and without children, respectively. Inconsistent attitudes or behavior was associated with elevated risk ratings for imminent re-victimization among women with children, whereas inadequate support or resources was associated with such risk among women without children. As previously discussed, women with children may be more likely to be living together with the perpetrator, and their display of inconsistent behavior or attitudes towards leaving the abusive partner could render the police to assess the risk for imminent IPV re-victimization as higher among these women, as they are still in contact with the perpetrator. In terms of the latter, this factor also approached significance for women with children indicating that inadequate support or resources is perceived by the police as an important factor for IPV re-victimization among victims in general. Being unable or unwilling to seek help from the police, or from family and friends, means that the perpetrator can continue to abuse the partner with little risk of the IPV being disclosed, which could explain why the police perceive this vulnerability factor as important for IPV re-victimization in general. Lastly, inadequate support or resources was also found to be more important for an elevated risk for severe or lethal IPV for women with children. It may be perceived by the police that perpetrators who use violence in the presence of children, in a setting where the family is isolated and disclosure and detection of violence are less likely, also may escalate their violence in terms of severity as well as being violent also against the children. Thus, the additional risk for violence against children in such a vulnerable context may explain the importance of this vulnerability factor for an elevated risk for severe or lethal IPV.

Finally, the police were more likely to recommend more than standard levels of protective actions in IPV cases with children present, including restraining orders, protecting a victim’s identity, and providing the victim with an emergency telephone. The emergency telephone could be a way of reducing the woman’s fears, while a restraining order and protecting a victim’s identity could avert potentially dangerous situations. The emergency phones distributed by the Swedish police are equipped with GPS functionality and a recording unit for telephone calls. As such, if the perpetrator approaches or calls the victim, the police can immediately respond to the victim’s location and/or gather evidence from the recordings. Noteworthy, the IPV reported to the police in cases with children present was less severe than in cases without children present, indicating that decisions on risk management may to a greater extent be governed by the presence of children rather than reported violence severity. This could also explain why protected living was not more often recommended to women with children, given their more unsafe living situation. Protected living, often at a women’s shelter, is an intrusive action that may harm the children’s wellbeing in the form of loss of social network and relationships (e.g., Vass & Haj-Yahia, 2020). Moreover, although women’s shelters are advocated for victims in Sweden, this protective action is usually recommended for the most severe IPV cases. This recommendation is also dependent on the victim’s attitude towards receiving help, where the high frequency of inconsistent attitudes and behavior among victims in our sample could have resulted in fewer recommendations of protected living. Possibly, women with children may be reluctant to accept protected living as they could perceive this to impact themselves and their children negatively, whereas a restraining order or emergency telephone may be perceived by victims as reasonably intrusive measures in their everyday life.

Nonetheless, due to our study design, we cannot draw any causal conclusions that the decisions on risk management strategies were due to the presence of children. To this end, more research about police decision-making in relation to risk assessment and risk management is needed (Robinson et al., 2018), especially in relation to the presence of children (Saxton et al., 2020). Yet, the results indicate that the police do discriminate between women with and without children, respectively when it comes to risk management of IPV. The tendency to recommend more than standard levels of risk management in IPV cases with children may be because the police perceive these cases to have more potential victims. These results contrast with previous studies that have suggested that police officers do not perceive children as potential victims (Richardson-Foster et al., 2012; Saxton et al., 2020). However, such studies have mainly examined police responses at the scene of the IPV incident, whereas the B-SAFER assessments in this study were conducted after the incident (i.e., not at the scene).

Findings in relation to the risk management strategies recommended by the police in this study are partially in line with previous research. First, the positive relationship between an elevated assessed risk for imminent re-victimization and more protective actions recommended by the police has previously been demonstrated (e.g., Belfrage & Strand, 2008; Storey & Strand, 2017). Second, extreme fear of the perpetrator was significantly more common among women with children. This finding is in line with Trujillo and Ross (2008), who concluded that fear of the perpetrator was the strongest predictor for more protective actions recommended by the police. Moreover, in line with our results concerning restraining orders, Trujillo and Ross (2008) also demonstrated that the victim’s level of fear of the perpetrator was a strong predictor for applying for an intervention order. As such, the victim’s fear of the perpetrator may strongly indicate an immediate danger that needs to be prevented, especially in the presence of children who are also exposed to the violence.

## Limitations

A major limitation of this study concerns the information available about the presence of children. As this was limited to their presence or absence, we do not know in what way they experienced the violence (i.e., witnessing IPV or being abused themselves). This precluded us from more detailed analyses of, for example, how victim vulnerability factors and police decision-making, in terms of risk assessment and management, differed across various forms of violence exposure or in relation to the number of children present in each case. Furthermore, our research design does not allow for causal conclusions about the impact of children on police decision-making in terms of risk assessment and management. Thus, although the results may indicate that the presence of children is considered by the police in assessing and managing risk for IPV, other possible confounders may account for the identified differences in this study. To this end, police decision-making in IPV cases has been found to be influenced by police officers’ perceptions of victims, perpetrators, and the credibility of reported IPV (El Sayed et al., 2020).

Information about the victim vulnerability factors was limited to their presence or absence. To this end, more knowledge about the specific content of each vulnerability factor would have been preferred. For example, health problems in the B-SAFER include several different types of problems that may impact the victim and the risk for re-victimization differently. As such, there is an important difference between having financial problems and mental health problems. Additionally, the results related to risk for re-victimization were based on the police assessments of such risk. Thus, future research could preferably examine to what extent these assessments are related to actual re-victimization experienced by the victims. Finally, the results found in this study should be generalized with caution as IPV reported to the police is usually more severe than, for example, IPV in community samples (NCCP, 2014; Sartin et al., 2006). Therefore, we do not know how well these results would extend to other samples of IPV. Also, the nature of the IPV in this study was limited to male-to-female perpetrated IPV meaning that generalizations to other relationship constellations, such as female-to-male or same-sex perpetrated IPV, cannot be made.

## Implications for Policy and Practice

The findings offer implications for policy and practice. The high number of children in this sample who have experienced IPV indicates that the police need to address and identify this group of victims, to make sure they receive protection and support. Related to both the Swedish Social Services Act (2001:453) and the Convention on the Rights of the Child, children have a right to be protected from violence and to grow up in a healthy environment that promotes a positive development, and this responsibility falls on the government, authorities, and adults in general. For this reason, children need to be viewed as victims of IPV. To this end, as of 2021, the use of IPV in front of one’s children is a punishable offense in Sweden (see the Swedish Government, 2021). Problematically, most IPV risk assessment instruments, including the B-SAFER, do not explicitly focus on the presence of children. Importantly, children are not just passive witnesses to the violence, they can be an active part of it, making the family situation and risk assessment complex (Saxton et al., 2020). Nevertheless, a stronger focus on the presence of children in relation to risk assessment and management of IPV is needed, both from criminal justice professionals (e.g., the police) and researchers.

To illustrate the importance of including the presence of children in IPV risk assessments, this study demonstrated that women with and without children, respectively, displayed different vulnerability factors and therefore have different needs. Thus, it is important that risk management strategies for these victims include the possibility to arrange safe housing options (e.g., women’s shelters). However, empowering women with children to separate and move away from an abusive partner is often a complicated task. The extreme fear of the perpetrator may make these victims more likely to return to the perpetrator to avoid an escalation of the violence. Moreover, some women have expressed that they want to keep their families together, that they are afraid of the justice system, and have an overall fear of the unknown that is associated with separating from their partner (Rhodes et al., 2010; Zink et al., 2003). Also, victims with children may in many cases be financially unable to move from their partner (Estrallado & Loh, 2014). To this end, it is important that victim support services to women with children include financial support from social services, enabling them to leave their unsafe living situation.

On a final note, the high prevalence of victim vulnerability factors among women in the overall sample highlights the importance of identifying victim-related characteristics when assessing risk for IPV re-victimization. The extent of psychosocial and behavioral vulnerabilities displayed by the sample further supports the recommendations to offer support and services to victims of IPV that target their psychological difficulties (Kuijpers et al., 2011). Considering that vulnerability factors are not in focus in most IPV risk assessment instruments, professionals tasked with assessing and managing the risk for IPV would receive a more appropriate and holistic perception about this risk if they also examined such factors (Belfrage & Strand, 2008).

## Conclusions and Future Research

The results of this study suggest that Swedish police officers conducting IPV risk assessments consider the presence of children in relation to the risk for a victim’s re-victimization and in their recommendations for risk management. Thus, women with children were assessed with a higher risk for being re-victimized and were recommended more than standard level of risk management strategies. These are encouraging results against a backdrop that depicts children as the forgotten victims in IPV cases (e.g., Reif & Jaffe, 2019). However, considering the large number of children who are exposed to IPV and its associated negative effects, it is important that a greater focus is placed on the presence of children in risk assessment and management of IPV. Drawing on the novelty of this study, as well as the cross-sectional study design, more research on children’s impact on risk for IPV re-victimization and police decision-making is needed.

## References

[CR1] Anderson K, Van Ee E (2018). Mothers and Children Exposed to Intimate Partner Violence: A Review of Treatment Interventions. International Journal of Environmental Research and Public Health.

[CR2] Belfrage H, Strand S (2008). Structured spousal violence risk assessment: Combining risk factors and victim vulnerability factors. International Journal of Forensic Mental Health.

[CR3] Bennett Cattaneo L, Goodman LA (2005). Risk factors for reabuse in intimate partner violence: A cross-disciplinary critical review. Trauma, Violence, & Abuse.

[CR4] Campbell MA, Gill G, Ballucci D (2018). Informing police response to intimate partner violence: Predictors of perceived usefulness of risk assessment screening. Journal of Police and Criminal Psychology.

[CR5] Capaldi DM, Knoble NB, Shortt JW, Kim HK (2012). A systematic review of risk factors for intimate partner violence. Partner Abuse.

[CR6] Carlson MJ, Harris SD, Holden GW (1999). Protective orders and domestic violence: Risk factors for re-abuse. Journal of Family Violence.

[CR7] Cater ÅK, Miller LE, Howell KH, Graham-Bermann SA (2015). Childhood Exposure to Intimate Partner Violence and Adult Mental Health Problems: Relationships with Gender and Age of Exposure. Journal of Family Violence.

[CR8] Council of Europe (COE). (2011). *Council of Europe Convention on preventing and combating violence against women and domestic violence*. Retrieved on 24^th^ of May 2021 from: https://www.coe.int/en/web/istanbul-convention/home?

[CR9] El Sayed, S. A., DeShay, R. A., Davis, J. B., Knox, K. N., & Kerley, K. R. (2020). A blue step forward: An exploratory study of law enforcement perceptions of intimate partner violence in the southern United States. *Journal of Interpersonal Violence,* 1-21. 10.1177/0886260520966675 10.1177/0886260520966675 33084493

[CR10] Erez, E. (2002). Domestic violence and the criminal justice system: An overview. *The Online Journal of Issues in Nursing, 7*(1). 12044215

[CR11] Estrellado A, Loh J (2014). Factors Associated With Battered Filipino Women’s Decision to Stay in or Leave an Abusive Relationship. Journal of Interpersonal Violence.

[CR12] Fantuzzo JW, Fusco RA (2007). Children’s direct exposure to types of domestic violence crime: A population-based investigation. Journal of Family Violence.

[CR13] Fernández-González L, Calvete E, Orue I, Mauri A (2018). Victims of Domestic Violence in Shelters: Impacts on Women and Children. The Spanish Journal of Psychology.

[CR14] Henderson AJZ, Bartholomew K, Dutton DG (1997). He loves me; He loves me not: Attachment and separation resolution of abused women. Journal of Family Violence.

[CR15] Holt S, Buckley H, Whelan S (2008). The impact of exposure to domestic violence on children and young people: A review of the literature. Child Abuse & Neglect.

[CR16] Kropp PR (2008). Intimate partner violence risk assessment and management. Violence and Victims.

[CR17] Kropp, P. R., Hart, S. D., & Belfrage, H. (2010). *Brief spousal assault form for the evaluation of risk (B-SAFER, 2nd ed.)*. User manual. Vancouver: Proactive Resolutions.

[CR18] Kuijpers KF, van der Knaap LM, Lodewijks IAJ (2011). Victims’ influence on intimate partner violence revictimization: A systematic review of prospective evidence. Trauma, Violence, & Abuse.

[CR19] Laing L (2017). Secondary Victimization: Domestic Violence Survivors Navigating the Family Law System. Violence against Women.

[CR20] Letourneau N, Young C, Secco L, Stewart M, Hughes J, Critchley K (2011). Supporting mothering: Service providers' perspectives of mothers and young children affected by intimate partner violence. Research in Nursing & Health.

[CR21] National Council for Crime Prevention (NCCP). (2014). *Brott i nära relationer: En nationell kartläggning* (Brå-rapport 2014:8) [Offences in close relationships: A national survey]. Retrieved on 7th of July 2018 from http://www.bra.se/download/18.9eaaede145606cc8651ff/1399015861526/2014_8_Brott_i_nara_relationer.pdf

[CR22] National Council for Crime Prevention (NCCP). (2021)*. Konstaterade fall av dödligt våld: En granskning av anmält dödligt våld 2020* (Brå-rapport) [Confirmed cases of deadly violence: A review of all reported cases of deadly violence 2020]. Stockholm: National Council for Crime Prevention.

[CR23] Nicholls TL, Pritchard MM, Reeves KA, Hilterman E (2013). Risk assessment in intimate partner violence: A systematic review of contemporary approaches. Partner Abuse.

[CR24] O’Leary DK (1999). Psychological abuse: A variable deserving critical attention in domestic violence. Violence and Victims.

[CR25] Petersson, J., Strand, S.J.M., & Selenius, H. (2019). Risk Factors for Intimate Partner Violence: A Comparison of Antisocial and Family-Only Perpetrators. *Journal of Interpersonal Violence 34*(2) 219–239. 10.1177/0886260516640547 10.1177/0886260516640547 27021731

[CR26] Reif K, Jaffe P (2019). Remembering the forgotten victims: Child-Related themes in domestic violence fatality reviews. Child Abuse & Neglect.

[CR27] Rhodes KV, Cerulli C, Dichter ME, Kothari CL, Barg FK (2010). “I Didn’t Want To Put Them Through That”: The Influence Of Children on Victim Decision-making in Intimate Partner Violence Cases. Journal of Family Violence.

[CR28] Richardson-Foster H, Stanley N, Miller P, Thomson G (2012). Police intervention in domestic violence incidents where children are present: Police and children’s perspectives. Policing and Society.

[CR29] Robinson AL, Pinchevsky GM, Guthrie JA (2018). A small constellation: Risk factors informing police perceptions of domestic abuse. Policing and Society.

[CR30] Sartin RM, Hansen DJ, Huss MT (2006). Domestic violence treatment response and recidivism: A review and implications for the study of family violence. Aggression and Violent Behavior.

[CR31] Saxton MD, Jaffe PG, Dawson M, Olszowy L, Straatman A-L (2020). Barriers to police addressing risk to children exposed to domestic violence. Child Abuse & Neglect.

[CR32] Social Services Act. (2001:453). *Socialtjänstlag, SoL*.

[CR33] Stöckl H, Devries K, Rotstein A, Abrahams N, Campbell J, Watts C, Garcia-Moreno C (2013). The global prevalence of intimate partner homicide: A systematic review. The Lancet.

[CR34] Storey JE, Strand S (2017). The Influence of Victim Vulnerability and Gender on Police Officers’ Assessment of Intimate Partner Violence Risk. Journal of Family Violence.

[CR35] Storey JE, Kropp RP, Hart SD, Belfrage H, Strand S (2014). Assessment and management of risk for intimate partner violence by police officers using the Brief Spousal Assault Form for the Evaluation of Risk. Criminal Justice and Behavior.

[CR36] Strand S, Storey JE (2019). Intimate partner violence in urban, rural, and remote areas: An investigation of offense severity and risk factors. Violence against Women.

[CR37] Strand, S., Petersson, J., Fröberg, S., & Storey, J. E. (2016). Polisens arbete med riskbedömning och riskhantering av partnervåldsrelaterad brottslighet: Implementering och utvärdering av införandet av strukturerade riskbedömningar för partnervåldsrelaterad brottslighet som en arbetsmetod vid polismyndigheterna i Västernorrland och Jämtland under perioden 2011-2014. [Police risk assessment and management of intimate partner violence-related criminality: Implementation and evaluation of structured violence risk assessments within the police districts of Västernorrland and Jämtland during 2011-2014]. Umeå: Brottsoffermyndigheten.

[CR38] The Swedish Government. (2021). *Lagrådsremiss - Barn som bevittnar brott [Law Council referral - Children that witness crimes]*. Stockholm: Government Offices.

[CR39] Trujillo MP, Ross S (2008). Police response to domestic violence: Making decisions about risk and risk management. Journal of Interpersonal Violence.

[CR40] United Nations (UN) (2006). World report on violence against children.

[CR41] Vass A, Haj-Yahia MM (2020). “Which home are we going back to?” Children's lived experiences after leaving shelters for battered women. Children and Youth Services Review.

[CR42] World Health Organization (WHO) (2013). Global and regional estimates of violence against women: Prevalence and health effects of intimate partner violence and non-partner sexual violence.

[CR43] World Health Organization (WHO) (2015). The health-systems response to violence against women. The Lancet.

[CR44] Yakubovich AR, Stöckl H, Murray J, Melendez-Torres GJ, Steinert JI, Glavin CEY, Humphreys DK (2018). Risk and Protective Factors for Intimate Partner Violence Against Women: Systematic Review and Meta-analyses of Prospective-Longitudinal Studies. American Journal of Public Health.

[CR45] Zink T, Elder N, Jacobson J (2003). How Children Affect the Mother/Victim's Process in Intimate Partner Violence. Archives of Pediatrics & Adolescent Medicine.

